# Challenges and needed reforms in midwifery and nursing regulatory systems in India: Implications for education and practice

**DOI:** 10.1371/journal.pone.0251331

**Published:** 2021-05-14

**Authors:** Kaveri Mayra, Sabu S. Padmadas, Zoë Matthews

**Affiliations:** University of Southampton, Southampton, United Kingdom; Queensland University of Technology, AUSTRALIA

## Abstract

**Background:**

In India, nursing regulation is generally weak, midwifery coexists with nursing, and 88% of nursing and midwifery education is provided by the private health sector. The Indian health system faces major challenges for health care provision due to poor quality, indeterminate regulatory functions and lack of reforms.

**Methods:**

We undertook a qualitative investigation to understand midwifery and nursing education, and regulatory systems in India, through a review of the regulatory Acts, and an investigation of the perceptions and experiences of senior midwifery and nursing leaders representing administration, advocacy, education, regulation, research and service provision in India with an international perspective.

**Results:**

There is a lack of importance accorded to midwifery roles within the nursing system. The councils and Acts do not adequately reflect midwifery practice, and remain a barrier to good quality care provision. The lack of required amendment of Acts, lack of representation of midwives and nurses in key governance positions in councils and committees have restrained and undermined leadership positions, which have also impaired the growth of the professions. A lack of opportunities for professional practice and unfair assessment practices are critical concerns affecting the quality of nursing and midwifery education in private institutions across India. Midwifery and nursing students are generally more vulnerable to discrimination and have less opportunities compared to medical students exacerbated by the gender-based challenges.

**Conclusions:**

India is on the verge of a major regulatory reform with the National Nursing and Midwifery Commission Bill, 2020 being drafted, which makes this study a crucial and timely contribution. Our findings present the challenges that need to be addressed with regulatory reforms to enable opportunities for direct-entry into the midwifery profession, improving nursing education and practice by empowering midwives and nurses with decision-making powers for nursing and midwifery workforce governance.

## Background

Midwives and nurses are integral to sexual, reproductive and maternal health care provision and are the primary health care providers in India. A comprehensive and strong regulatory mechanism is therefore needed to regulate education, practice and to ensure competent nurses and midwives in the Indian health workforce. The International Confederation of Midwives (ICM) recommends six functions that regulatory bodies should maintain; setting the scope of practice, pre-registration of education, registration, re-licensing and continuing competence, complaints and discipline, code of conduct and ethics [1]. The Indian Nursing Council (INC) and State Nursing Councils (SNC) play key roles in the regulation of nursing and midwifery education in India. They oversee registration, licensing, inspection and examination. However, there is duplication of these roles at the national and state levels [2].

In India, nurse-midwives become part of the health care workforce after completing 2–4 years of mandatory Pre Service Education (PSE) which aims to provide skills of both of the professions of nursing and midwifery. Hence, in this paper we address the existing cadre as ‘nurse-midwives’ although they are generally referred to as nurses. Currently, India does not have a cadre of competent independent midwives [3]. Midwifery education is provided both as a part of a diploma course called General Nursing and Midwifery (GNM) for three years and also as a part of a four year BSc nursing degree. The midwifery component in BSc nursing degree lasts for approximately six months. This compares to the longer duration of 18 months midwifery education elsewhere globally after completing three years of a nursing degree [4]. There are also elements of midwifery skills education embedded in the Auxiliary Nursing and Midwifery (ANM) certificate course. The curriculum for these three entry-level courses of midwifery education are not comparable with the ICM recommended skill-set [1]. However, those graduating with a GNM diploma or a degree in nursing are addressed as nurses or nurse-midwives. The National Health Mission (NHM) launched by the Government of India (GOI), recently initiated efforts to formulate operational guidelines to implement midwifery education [3]. An existing diploma course for Nurse Practitioners in Midwifery (NPM) has been updated to implement midwifery in India, though it is still not completely in line with the ICM recommendations for training competent midwives [3]. It is essential to understand and tackle the shortcomings of India’s midwifery education, regulation and practice capacities, especially considering that 83% of maternal deaths, stillbirths and neonatal deaths can be averted when care is managed by professionally trained midwives [5].

Midwives and nurses are often neglected and subjected to discrimination throughout their education and through to their professional careers [2, 6, 7]. Indian nursing and midwifery education is faced with several challenges including a lack of qualified teachers; a mismatch between theory and practice in learning; a lack of opportunities for practice; and gender-based discrimination and stigma [2, 7–11]. Around 88% of nursing education in India is provided by the private sector where the quality of education is reportedly poorer than that offered in public institutions, especially in the resource-poor large states of northern India [8]. The private share in nursing education has continued to grow in terms of the number of training institutions and recruitment quota, in response to the global demand of nurse-midwives, thus ranking India the second in terms of nurses’ outmigration [12]. The uneven distribution of nursing institutes is yet another challenge and the privatization of education has led to a skewed production of human resources for health (HRH) [8]. Education is concentrated in the six states of Andhra Pradesh, Karnataka, Kerala, Maharashtra, Pondicherry and Tamil Nadu, which represent only 31% of the Indian population yet have 63% of nursing and 58% of medical colleges [8]. This compares to to the eight low HRH-producing states of Bihar, Chhattisgarh, Jharkhand, Madhya Pradesh, Uttar Pradesh, Uttaranchal, Odisha and Rajasthan, which hold 46% of the total population in India, but have only 20% of nursing and 21% of medical colleges [8, 13]. The training institutes are further biased toward the urban areas within these states [8].

Regulatory mechanisms are found to be relaxed to allow training in certain private institutes, despite capacity challenges [9]. These issues are further reflected in the strength of the nursing workforce. On average, India has only 17 nurses and 9 doctors per 10,000 population [14], unevenly distributed between states, urban-rural and public-private sectors, with over 70% of the nursing workforce employed in the private sector [15]. The implementation of a good regulatory framework is therefore essential to address the outstanding issues in nursing and midwifery education and practice.

The current structure of midwifery and nursing regulation also faces many challenges in India. The system is dominated by male medical and non-professionals and provides little authority to nursing and midwifery regulators [10]. The role of gender in nursing and midwifery is not systematically understood in India [7, 10]. The regulation of nurses and midwives’ migration is unclear; the regulation of practice is weak, and there is a failure to improve the quality of education mainly in the private sector [8, 16–21]. Over the last few years, non-government and international development organizations have coordinated efforts to improve pre-service and in-service education in India [21, 22]. However, these efforts have not succeeded in establishing and sustaining a strong regulatory structure. There have been some initiatives focused on improving the quality of nursing and midwifery education and services in India, however, the challenges in regulation remain unaddressed [8, 11, 19].

The goal of this research is to understand the influence of current regulatory system on nursing and midwifery education, practice and development in India. More specifically, the paper has four interrelated objectives: (i) to document the regulatory system of nursing and midwifery in India through a review of the existing regulatory acts; (ii) to understand the challenges and weaknesses in the system of nursing and midwifery education and practice regulation; (iii) to investigate the gender and power based issues underlying the regulatory challenges of midwifery and nursing and (iv) to document the challenges of midwifery education and practice under nursing regulation and governance.

## Materials and methods

### Study design

This research adopted a qualitative design involving in-depth interviews to examine the perceptions and experiences of nurse-midwives in senior leadership roles regarding the regulatory systems of midwifery and nursing in India. The study participants were initially selected through a purposive sampling based on contacts with the lead researchers in the field, followed by a snowball sampling in five states in India: Rajasthan, Odisha, Bihar, Madhya Pradesh and West Bengal and at the national level. The five states selected represent different cultural, social, economic and health contexts. All selected states, except West Bengal, are amongst the low HRH-producing states with poor quality of midwifery and nursing education. West Bengal is widely acknowledged for providing good quality nursing and midwifery education and governance, especially in the public sector [23]. The inclusion criteria for in depth interviews followed a strategy to select nursing and midwifery leaders who represent various domains of administration, advocacy, education, regulation and service provision in the selected states and at the centre. Some of the participants represented multiple domains. We interviewed selected leaders at the national level to understand the larger context of health policy making and nursing governance in India. In addition, we reviewed the nursing and midwifery regulatory acts from all the five selected states and the Indian Nursing Council Act to understand and compare the guiding documents and protocols for regulation. The review is summarised in the results section.

To understand the global experiences and perspectives on the regulatory frameworks of nursing and midwifery, we conducted interviews with eight international experts representing various international organizations that play a key role in guiding the health policy and regulatory frameworks globally. We interviewed senior nursing and midwifery professionals from the World Health Organisation (WHO) headquarters, International Nursing Council (ICN), International Confederation of Midwifery (ICM), United Nations Fund for Populations Activities (UNFPA) and senior midwifery academics from three research intensive higher education institutions in the UK.

### Data collection and analysis

In-depth interviews were conducted between July 2018 and January 2019. The participants were informed about the purpose of the study by email or phone before seeking formal appointment for interviews. We approached 43 nursing and midwifery leaders, and of these nine could not participate due to their prior commitments. All the interviews were conducted in person, except three which were done via video conference call. Each participant was interviewed only once. We followed a semi-structured questionnaire guide which included three sections: 1) background information of participants including their education relevant to nursing and midwifery, professional experiences, roles and responsibilities; 2) current and past role and responsibilities of participants in nursing and midwifery regulation and governance and 3) participants’ reflections of nursing and midwifery regulations including their perceptions on the state of education and practice regulation.

All interviews were conducted by the lead author, an experienced qualitative researcher with educational background in nursing, midwifery, public health and with research experience on issues pertaining to nursing and midwifery in India. All participants were aware of the lead researcher’s background, professional qualifications and the rationale of the study. The interviews were carried out in English language in most cases, or in Hindi and Bengali in a few states where the transcripts were translated into English. The female lead researcher is fluent in these languages. Most interviews were conducted in the work place of the participants, except for a few at their homes or in a public place. Each interview lasted approximately for little more than an hour depending on content and information saturation. Data were processed and analysed thematically using NVivo 12 software. The lead researcher conducted coding simultaneously with data collection which helped to clarify emerging themes in subsequent interviews. The codebook consisted of apriori codes which were supplemented by deductive codes, as the analyses progressed. Relevant codes were grouped into themes such as gaps in regulatory functions, gender roles on regulation and other key emergent issues.

### Ethical considerations

The study obtained formal ethical clearance from the University of Southampton Faculty of Social Sciences Research Ethics Committee of the Authors’ institution prior to the start of the research. All participants read and signed the written consent prior to data collection. All respondents were provided with a participant information sheet with details about the research. The researcher sought formal permission to audio record the interviews. Three interviews were not audio recorded since the participant refused consent, instead written notes were taken. To ensure confidentiality, study respondents were anonymised throughout the analysis and presentation of results.

## Results

The interviews focused on the quality of nursing and midwifery education, regulation, challenges of regulation in education and practice, and recommendations to improve regulation of nursing and midwifery education and service provision.

The age of respondents varied from 46 to 83 years. All respondents were midwives and nurses except one who was not a midwife. Twenty-six participants completed their education in nursing and midwifery in India. Their qualification was the degree that combined nursing and midwifery curricula. Four out of the 34 participants were men, who were all interviewed in Rajasthan, one of the few Indian states where men have historically been permitted to opt for midwifery and nursing education. All respondents held senior level positions, except for two who had retired from service. The total experience of the participants ranged between 24 to 60 years. Table 1 presents relevant demographic qualifications and work profile related information of the participants.

**Table 1:** Participant profile.

Indicator	No. of participants/ Range	Number of participants by response to each question
**Age Range**		
40–60 years	18	30
61–80 years	11	30
>80 years	1	30
**Gender**		
Female	30	34
Male	4	34
**Qualification**		
RN, RM	32	33
RM	1	33
MSc Nursing and MPH	22	33
PhD	11	33
**Experience (years)**		
Urban Experience Range	5–46	17
Rural Experience Range	0–25	17
Total Experience Range	17–60	29

### The regulatory system of midwifery and nursing in India: A review of Acts

This section presents a review of the regulatory acts implemented in the selected states and centre (Table 2). All the acts are extracted from the respective council’s website.

**Table 2:** Nursing and midwifery Acts reviewed, by year of enactment.

Name of the Act	Year of enactment
The Indian Nursing Council Act [24]	1947
The Bengal Nurses Act [25]	1934
The Bihar and Orissa Nurses Registration Act [26]	1935
The Central provinces Nurses Registration Act (Madhya Pradesh) [27]	1936
The Orissa Nurses and Midwives Registration Act [28]	1938
Rajasthan Nurses, Midwives, Health visitors and Auxiliary Nurse Midwives Registration Act [29]	1964

As can be seen from Table 2, most of the state Acts are older than the INC Act of 1947. All the state Acts are similar in content which includes the profile of members, key definitions, membership conditions, information for professional registration, re-registration and service related clauses for practitioners. The membership of the governing body is not uniform across the Acts, ranging from seven members in Odisha to fifteen in Bihar. Every council has a set number of members who are doctors and some members are non-nursing/ midwifery administrators. The ex-officio members, four to seven in every council, can be elected multiple times as long as they hold the position by virtue of which they have been elected. There is no system of direct application unless through nomination, followed by election by members. None of these Acts have been re-created according to the needs of the states, neither have they been adequately amended since they were introduced in the 1930’s and 40’s despite immense changes in the health system and the population. Bihar shared a council with Odisha at the time of its creation (in 1935) and mentions ‘Orissa’ in the title even though Odisha started a separate council shortly afterwards (1938).

The language of the Acts is not gender sensitive and all the Acts refer to the registrar as ‘he’ or ‘his’ despite, historically, the position of registrar in most nursing councils being held by women, including at the time of this study when four out of five SNC’s registrars in the study states are women. The curriculum is not part of the Acts, and is centrally designed by INC and implemented with some variation in the states.

The content varies a little for some Acts. The Rajasthan Nurses, Midwives, Health visitors and Auxiliary Nurse Midwives Act of 1964 is the most detailed Act entrusting the power of council through eight activities, including grounds on which the state government has the right to dissolve the Council and the Act. The language of the RNC Act is relatively gender sensitive. Odisha and Rajasthan’s Act and Council includes ‘midwives’ in the title. None of the other Acts mention independent midwifery practice. Odisha is the only state that registers *dais* (traditional birth attendants). The Central Provinces Nurses Registration Act of 1936, in Madhya Pradesh discourages private practice, although it does elaborate on it to clarify what it means for nurses working in the private health care provision and education sector.

All six Acts mention regulating education, but none highlight regulating practice or updating the knowledge and skills of practicing professionals. There are no separate Acts at the state or centre that regulate nursing and midwifery practice. The Acts do not mention INC’s role in supervising the SNCs. The accountability mechanism between the INC and the SNCs is unclear. This could be because the state Acts, except Rajasthan, were formed before the INC Act of 1947—although Rajasthan’s Act also does not mention INC’s role. INC’s key activities focus on maintenance of registers for all nursing and midwifery courses, registration at the national level, licencing of nursing training institutes, setting the curriculum for every course and maintaining uniformity. The INC website has information on the National Registration Tracking System (NRTS), which was recently launched to maintain a database of nurse-midwives in the country from every state, to enable tracking and to regulate placement. As of August, 9^th^ 2020, there were 9, 90,524 professionals enrolled under the NRTS (INC, 2020).

The council Acts do not provide any guidance on nurses’ domestic or overseas migration clarifying the terms of registration while serving in a foreign country, or their practice in India on return, higher education in nursing and midwifery or other health-related education in other countries.

### Challenges and weaknesses in the regulatory and governing bodies

The INC regulates nursing and midwifery education in India. The SNCs manage regulation in the respective states. Regulation of nursing and midwifery education covers certificate, diploma and degree courses in the public and private sector. Every council has positions of President and Registrar as the key administrators. Routine administration is in the purview of the Registrar. One participant from the centre objected to the processes and terms of reference of administrators at the councils.

“*These days in nursing council a person can be President for life*! *The council seems to be happy with it… elections are conducted in every term but the leadership does not change*. *They can change that if they want to*.*”* (National)

“*INC president has been in position for 15 years… One term of leadership at INC is 4 years*.*”* (National)

Most council participants mentioned a lack of human resources as a key challenge to managing the councils work such as admission, examination, inspection, registration and re-registration in each state. The role of the INC is different from the SNCs. The INC sets the national curriculum, oversees registration, implements the NRTS and conducts inspections in all the states. Some of these services overlap, such as institutional inspections which are carried out both by the SNCs and the INC independently to start and maintain a new institution. The reason for this was not clearly explained by the participants. One respondent from Rajasthan commented that this duplication of activity was unnecessary and should be handled solely by the respective SNCs.

Workload challenges were repeatedly mentioned particularly because the number of training institutes are increasing rapidly. Table 3 shows the number of nursing and midwifery educational institutes in each study state (along with seats) and total institutions in India. Between 2005 and 2018, the total percentage increase for ANMTCs is 516% (254 to 1564), for GNMTCs it is 187% (979 to 2812) and for colleges of nursing its 405% (349 to 1761). Bihar has the lowest number of institutes and admission capacity, while Madhya Pradesh has the highest. Bihar has 8.6% of India’s population but only 0.5% of total colleges of nursing. The capacity of Bachelor’s degree in nursing education has increased four times and post-graduate studies in nursing by eleven times in India between 2005 and 2018. This increase has been disproportionate. The number of institutes providing GNM education increased significantly from 22 to 324 in Madhya Pradesh but remained low in Bihar (13 to 21) between 2005 and 2018. The number of GNM places for admission is disproportionately high, ranging from 991 to 12,970, especially given that the population coverage is highest in Bihar followed by West Bengal, Madhya Pradesh, Rajasthan and Odisha.

**Table 3:** Nursing and midwifery training institutions in selected states (2018).

State	Year	BSc Nursing	MSc Nursing	General Nursing and Midwifery	Auxiliary Nursing and Midwifery	Population (2011 Census)
**Bihar**	2018	9	0	21	90	10,38,04,637
	2011	0	0	11	29	
	2005	0	0	13	25	
**Madhya Pradesh**	2018	140	50	324	71	7,25,97,565
	2011	101	29	135	82	
	2005	19	2	22	9	
**Rajasthan**	2018	169	26	164	12	6,86,21,012
	2011	149	5	176	11	
	2005	3	0	57	8	
**Odisha **	2018	29	11	78	127	4,19,47,358
	2011	16	5	48	67	
	2005	4	0	10	16	
**West Bengal **	2018	23	12	72	6	9,13,47,736
	2011	16	7	49	59	
	2005	2	2	28	20	
**All India **	2018	1,761	590	2,812	1,564	1,21,08,54,977
	2011	1,570	450	2,351	935	
	2005	349	54	979	254	

Data source: Indian Nursing Council Website, Census 2011.

Bihar does not provide any higher education opportunities at postgraduate level for its graduating nurse-midwives in the state, thus restricting the growth of educators to impart degree level education. Each of these states have 20–100 seats for GNM and BSc Nursing per institute. The number of training institutions are not proportionate with the state’s population. The INC does not disaggregate the distribution of institutes and admission capacity by public and private sector.

The regulatory challenges in education are different in public and private institutions. Although the curriculum being taught is uniform in every state, the respondents argued that quality of education is not the same in the public and private sector. Respondents from every state shared that regulation of education was comparatively poorer in private sector institutions.

*“Practical experience* (for students) *is zero in private sector”* (Bihar)

“*Private sector regulation is poor*. *No one sees that*.*”* (Bihar)

Health facilities have affiliations to both public and private medical and nursing education institutes. It is difficult for the hospital authority/ staff to ensure that every student receives the required amount of practice as recommended for successful course completion. A respondent from Bihar shared her experience of working in a government tertiary level teaching hospital:

“*Head of the Department* (doctor) *says my medical students will practice first* (in the labour room). *The nursing* (and midwifery) *students observe cases but can only request to give them a chance to practice*. *100% cases* (births) *are conducted by medicine students… the council inspected*, *yet did nothing to change this*.*”* (Bihar)

Such issues were shared by participants from all states, except West Bengal. The most common challenge mentioned was students from private institutions not getting an opportunity for practical experience during pre-service education. Data suggests that students often filled up their case books with fabricated cases as a way to pass the course. This practice goes unchecked, though well acknowledged. Even more alarming is the illegal procurement of fake certificates by untrained persons. To address this issue, the councils take precautions before registering candidates from other states. However, the Registrars do not have sufficient resources to tackle such challenges which represent a major barrier in councils’ functioning.

The lack of practical exposure for students in private institutes leads to a lack of knowledge and skills in comparison to those from government-led institutes. This challenge is acknowledged in private hospitals. Most private hospitals have their own education institutes but reportedly they do not have confidence in their own students because of concerns over their lack of skills. A participant from Rajasthan reflected on the poor training quality of students from private institutes and acknowledged that the state council is aware of the problem.

“*The* (state) *council knows about it and does nothing”* (Rajasthan)

The participant further shared that students sometimes pay bribes to their instructors invigilating practical examinations or even bribe by inserting money inside their answer sheets during examination for attracting good credits for their theory papers. Many teachers succumb to this practice, but not all surrender to the pressure as mentioned by a participant:

“*No one fails students… it is all hidden*. *Student goes to drop the examiner at the train station to pass on an envelope*. *I have never taken that envelope*. *I have heard 5000 rupees is minimum per student for BSc and GNM… Everyone wants to be an examiner for private institute*, *for that extra income and no one wants to go to government institutes cause government students won’t pay to pass*.*”* (Rajasthan)

Corruption is the underlying reason for such malpractice which is kept in place by promoting nurses who are party to it.

“*When nurses raise their voice*, *government removes them from their position*. *They are not scared of us as we don’t have any power*. *We are dominated from above*. *We know everything but can do nothing*” (Rajasthan)

**Fig 1:**
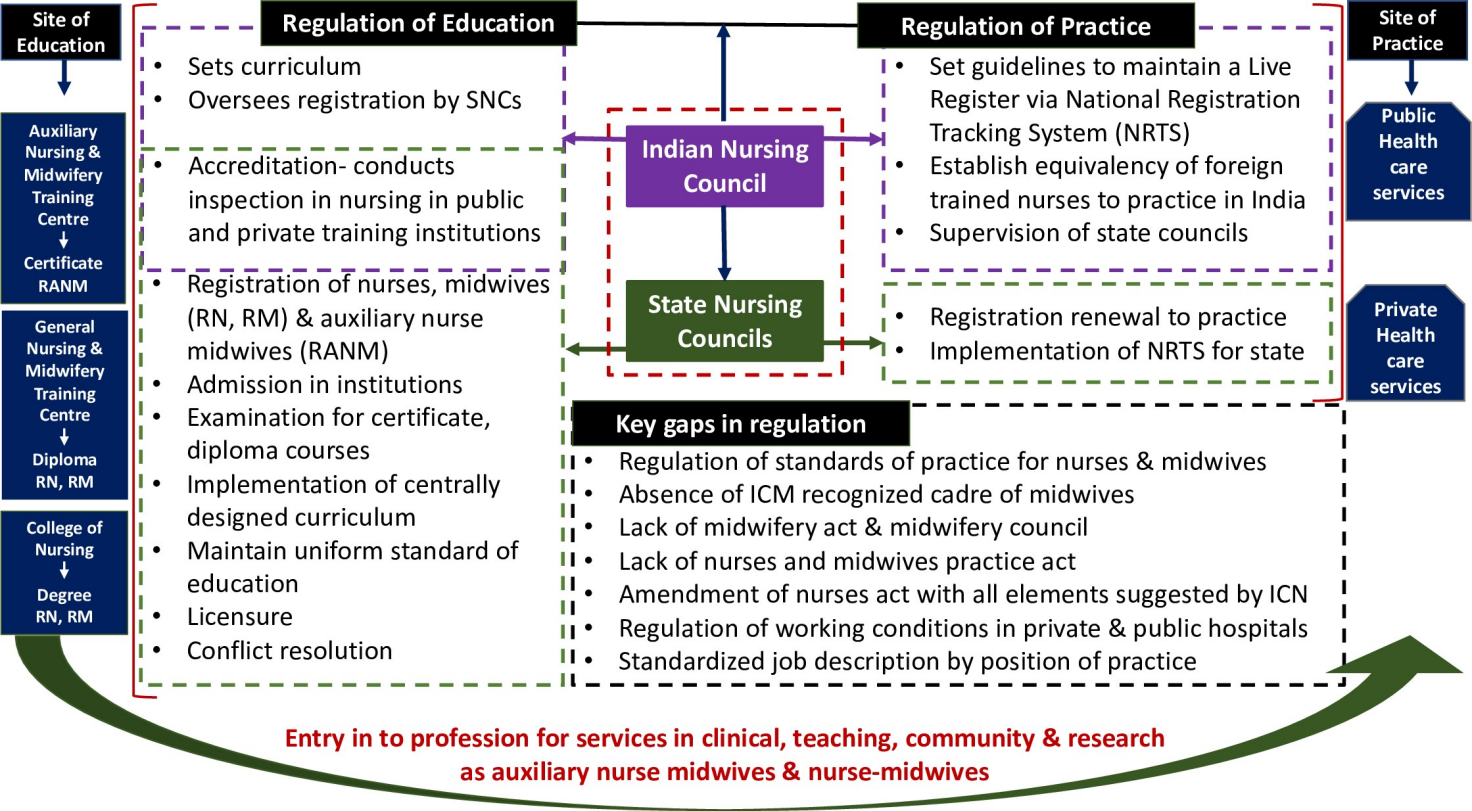
INC and SNC’s role and gaps in regulation of nursing & midwifery in India *(author’s original)*.

A participant from Madhya Pradesh commented on the issue of student non-attendance. Instead of sitting through the classes, students work in smaller nursing homes as assistants for an extra income. These training centres are usually affiliated to big private hospitals, so in terms of requirement, their *‘papers’* are always complete which means the non-attendance goes undocumented. Even though these nursing institutes undergo inspections from the SNC, INC and the state government, they often manage to overcome any regulatory actions. Fig 1 shows the current responsibilities of state and central council, along with the overlap in their role and the gaps in regulatory functions.

### Gender and power influencing midwifery and nursing regulation

The nursing and midwifery professions face some unique gender-based challenges. In a female dominated profession, the leadership in regulation is male dominated. In Rajasthan, the curriculum is regulated under the leadership of a male nursing registrar and practical experience is overseen by a male nursing administrator from the state health ministry. Rajasthan and Madhya Pradesh are amongst the few states that allow male candidates to take up nursing and midwifery education. Although, Rajasthan historically had both male and female candidates in diploma and degree level courses, fewer women opt for nursing careers due to professional stigma associated with nursing and midwifery. The difficulty to ensure the required midwifery practical experience for male candidates has been a persistent challenge. In some institutions, the professor or clinical instructor in charge of midwifery is a male, who is unlikely to have ever assisted a single birth. The issue of the lack of practical midwifery education for male candidates has not been addressed. A participant from Rajasthan, who teaches midwifery, raised this issue faced during his own education:

“*I asked them why are you giving us this training when you won’t let us practice during the training*. *What is the point of doing this training*?… *They* (regulatory bodies) *are not even thinking in those lines*.” (Rajasthan)

The INC recommends for students to assist 25 births each in BSc Nursing and MSc Nursing in Obstetrics. A male participant who teaches midwifery assisted only five births including his BSc in Nursing and Masters in Obstetrics degrees, even though 650 hours of study is required in total for the post-graduate degree. Getting a chance to assist those five births was fraught with difficulties involving persistent efforts to win the trust of the labour room’s team of care providers.

“*There is stigma for men to work in labour room*. *Families don’t encourage it*, *so the scope is less*.” (Rajasthan)

“*This gender related problem has existed for over 30 years but INC is not doing anything to address it… the state nursing councils can not do anything about this*. *Now INC is implementing an 18 months course in midwifery but not looking into the challenges of men in midwifery… INC needs to take a stand*… *States can not do anything*. *INC tells*, *we do*. *State council can write to INC*, *but they don’t care about the quality of education”*. (Madhya Pradesh)

The nursing and midwifery leaders representing education, administration and service provision brought up similar issues regarding male students lacking practical midwifery exposure. However, the participants representing regulatory bodies shared no such concerns. West Bengal has recently started enrolling male candidates for nursing and midwifery education. The issue of gender is not just about getting a chance to practice midwifery. A participant mentioned that the apparent gender imbalance in the profession is also a reason for the lack of leadership for women in nursing and midwifery.

“*People in West Bengal used to think men in nursing won’t be accepted by society*, *but that was a myth*. *There are two colleges with 50 seats each for male candidates who are also learning midwifery*. *The 1*^*st*^ *batch training is on and it is very exciting*.” (West Bengal)

The role of doctors, who are usually men, is explored in different ways. They are held responsible for the lack of female representation and growth of the profession. There is frustration about doctors holding key positions in nursing councils.

“*Nursing association wants the nursing directorate to be separate so their demands can be addressed*. *Any demand from a nursing or midwifery association is usually shelved when a doctor policy-maker comes in the picture*.*”* (Rajasthan)

“*The president of Bihar Nursing Council is a Doctor…there is a lot of politics in all of this*. *There is pressure from the* (Health) *Secretary as well*.*”* (Bihar)

An interesting rationale came from a participant in West Bengal on the lack of leadership quality amongst nurse-midwives. According to her, the issue is that “*lesser doctors are falling in love with nurses”* as more women are being educated as doctors. Given that more recently male doctors are getting married to female doctors, nurses seem to be falling further down in the hierarchy of healthcare. The position of nurses tends to diminish over time as doctors do not consider them their equal anymore. Participants suggest that the involvement of men in nursing is deemed to uplift the image of nursing in the country to reduce the gender based stigma.

“A*s women*, *we are ruled by our father*, *brother*, *husband and son at different stages of our life… It is our lack of confidence and attitude that only if men are there will we succeed*. *There is a dependence… we surrender too easily*.*”* (West Bengal)

### Midwifery as a part of nursing: A regulatory challenge

Midwifery is usually practiced on rotation with other nursing roles, and is also not part of a direct entry education. Direct entry midwifery is a three year degree course recommended by ICM that provides a license of Registered Midwife (RM). Participants shared mixed opinions on the requirement and future of midwifery in India as an independent profession. While most participants seemed to be in favour of independent midwifery, there were limited and unclear responses on the regulatory challenges it entails. A respondent from Odisha could relate to working independently in the periphery and yet working harder, as the best phase of her career.

“*ANM is our independent midwifery practitioner who is assisting deliveries in rural areas as good as doctors are doing independently in the urban areas*. *Some ANM’s conduct deliveries much better than doctors and are very famous for their work*, *people specially request them to assist with their delivery*.*”* (Rajasthan)

Medical or obstetric domination is reported as a key barrier to independent midwifery practice as the respondent mentioned “*we can not work independently in the tertiary level as the* (medical) *professors are there”* or that “*we can not work without their permission”*. Several respondents mentioned alienation and exclusion as key issues in the tertiary level of care, although they have been entrusted with larger responsibilities at the primary and secondary levels.

“*I have done spinal anaesthesia*, *caesarean section and abortion*, *under supervision*. *If a policy is made that we can work independently*, *it will be uplifting for the profession* (of midwifery).*”* (Odisha)

“*Independent midwifery is key to address the situation with disrespect and abuse during childbirth everywhere*.*”* (Bihar)

“*Nurse and midwife should be separate cadres*, *like medicine*. *Rotation is not helping”* (National)

Another participant mentioned the lack of a legal framework as a key challenge for independent midwifery in India. This is due to a lack of legal protection for midwifery practitioners, unlike with doctors. At the national level, participants felt that the INC should take charge of regulating nursing and midwifery services. These challenges were echoed unanimously by all participants.

“*If the INC is the* (only) *regulatory body* (in the country) *then that should look after practice*. *In the 10 years that I have been* (Nursing) *superintendent*, *no one has come to check the competency level of my nurses”*. (National)

“*Nothing is happening in terms of nursing regulation*. *There is no regulation of service*.*”* (Bihar)

There are challenges of underfunding as well, which were identified by an international expert.

*“All of them* (councils) *are badly underfunded*. *INC has managed to get some funds but given the size of India*, *it’s peanuts*. *It would be effective if they had many more resources*. *They could really meet*, *coordinate*, *re-educate*, *train*, *get the evidence and really understand what’s going on*. *Its sad that what’s all happening at states is registering and re-registering*.*”* (International expert)

Respondents felt that the councils should work in favour of midwifery and protect midwives’ right to practice as an independent profession. The need for an exclusive midwifery regulatory Act was mentioned a few times. A participant stated that a Nursing and Midwifery Practice Act of India is being drafted without any assurance of when it will be enacted. Meanwhile, another participant commented that the lack of a midwifery model of care is due to the vested interests of national leaders “*… they do not want independent midwifery in India”*. The independent status of midwifery is expected to bring more recognition and a boost in salary as is seen in many other countries. Another international participant suggested a way forward:

*“I think it would change the status if the public sees that this is a midwife*, *this is her level of skills*. *Someone who practices independently*, *not dependent on doctor*. *It’s straightforward*. *It automatically shifts the status of the profession*. *It is fundamental to have that independent status*. *I know it’s not easy to organize and make happen*. *But it’s the way forward… Changes in policies will of course support the midwives but also part of what’s needed is to get midwifery leaders in the profession who sit there at those tables*. *There are policies being made about midwifery and maternity care without them at the table*. *We have got to get ourselves at those top tables… There is strong evidence on midwifery with The Lancet series*. *It doesn’t happen overnight*.*”* (International Expert)

## Discussion

The ICM identifies education, regulation and association as the three pillars for development and practice of midwifery [1]. The nursing and midwifery workforce in India faces many challenges in each of these three areas, especially poor quality of education stemming from a weak regulatory structure that needs to adapt to changes over time. The lack of leadership role and decision-making power for nurse-midwives’ further weakens the governance of these professions dominated by doctors [8, 10, 20, 30].

India does not have a professional midwifery workforce or direct entry midwifery education yet. Hence, regulation is currently targeted at nurses who are playing a dual role of nurse and midwife. As more evidence is generated on the advantages of midwifery for maternal and neonatal health [31], it becomes important for the INC to make legitimate efforts to start direct entry midwifery education that will create a cadre of midwives independent of their nursing role. The INC’s role will be fundamental in formulating the regulatory structure, which needs to be supported by the respective SNCs in generating evidence and ensuring that culturally appropriate changes are made. This could be done as an addition to the existing INC and the SNCs with appropriate amendments to the current acts, or by creating a separate midwifery council with a midwifery Act.

Our study has a few limitations. In November 2020, the GOI introduced the draft National Nursing and Midwifery Commission (NNMC) Bill [32]. The new bill is unlikely to address the existing issues, but instead may create additional challenges which include undermining the autonomy and independence of the state regulatory bodies by centralizing decision making, and not allowing diversity in representation in the commission membership and governance. When passed, the NNMC Bill may end the prospects of addressing the several regulatory challenges in nursing and midwifery education and practice in near future. This statement is being made in cognizance that changing regulations is a time consuming process evident from the fact that the upcoming bill will replace the INC Act of 1947 after 74 years. Our study participants included senior level leaders in nursing and midwifery within public institutions at the state level, and organisations/ associations representing public and private at the national level. It is possible that some of the responses based on personal observations or views could be biased. However, we were able to establish convergence in response patterns across participants from different states.

The Acts in the five states and the INC Act have not been appropriately amended for decades, and most of these Acts were created before independence. An independent review pointed out that INC Act addresses only 15 out of 21 general, structural and functional elements recommended by International Council of Nursing (ICN) [33, 34]. The current INC Act is mainly educational and lacks many functional elements such as ‘continuing training required’ under education and training; ‘code or standards or conduct/ ethics’, ‘disciplinary procedures’ under fitness to practice; ‘established process under appeals’; ‘offences/ penalties listed’ under offences; and funding of council [34]. The Acts also need to include key definitions such as nurse, nursing, midwife, midwifery and their specific scope of practice [34].

Interviewing leaders from different domains of nursing and midwifery governance helped identify many other challenges. The increasing workload was frequently mentioned by participants representing regulatory bodies, also raised as a concern in other countries by international experts [34]. The participants did not specifically mention the cause of discrimination in education, partial treatment of medical students in comparison to nursing and midwifery students and the powerlessness in nursing, midwifery and health policy making they faced as ‘gender-based’ even though it is clear from their responses. The doctor dominance and political nature of regulation was reported with caution. All these challenges and more, including lack of leadership qualities in the regulatory bodies, were raised by nursing and midwifery leaders representing education, service provision, association and administration. Respondents urged the need for more transparency and inclusivity in the regulatory processes of INC and SNCs. This is essential to ensure accountability [1, 20]. The participants felt that a change in leadership at INC might improve regulation and that the key position should not be stagnant.

The regulation of private education is particularly concerning. Note that 88% of India’s nursing education is being conducted in the private sector [8]. This creates avenues for corruption and lack of control [20]. There is a dire need for regulatory bodies to address the challenges related to non-attendance of students, working in the nursing homes during pre-service education, lack of qualified teachers, less opportunities to practice and illegal practice with no formal qualification.

The findings from this study confirms the evidence reported elsewhere, and reinforce the urgent need to improve midwifery and nursing education [30, 35–37]. In the Indian context, shortcomings in regulation have persisted for decades [8, 16–19]. The participants of this study have clearly identified that key stakeholders have failed to take a gender sensitive approach. The scope of workforce participation for people who identify as transgender or gender non-conforming, and the challenges they face as nurses and midwives, remains unexplored in India. Nursing and midwifery needs a people centric approach to address the existing gender-related barriers. Gender-based discrimination begins with each nursing and midwifery student’s education and extends to their clinical practice or teaching thereafter. When male candidates are given opportunities for graduation and post-graduation in midwifery (gynaecology and obstetrics) and nursing, the SNCs must ensure that they receive enough practical experience without any gender discrimination [16–18]. Men and women in the profession have specific issues that need to be addressed in a way that ensures quality education, opportunities to practice and provide care in line with patients’ rights and choices. Nursing and midwifery being traditionally women dominated professions, adds to the gender based discrimination and stigma, which gets exacerbated because nurse-midwives often come from poor socioeconomic background and backward classes and castes, which has a relevant history behind it on how nursing began and progressed in India [7, 10, 38]. Though it is established that these characteristics have an impact on nurses and midwives education and practice but the extent of it alongside the practice of medicine, owing to the intersectionality based on their personal attributes, remains to be studied in the context of India [7, 10, 39].

A key challenge is discrimination between nursing-midwifery and medical students, as they practice in the same health care delivery system. This discrimination results in inadequate practical experience opportunities for nursing and midwifery students, and establishes a hierarchy in the medical care system from pre-service education onwards. This clearly demonstrates the lack of attention afforded to nursing and midwifery education in comparison with medical education. This hierarchy is often gender-based and creates inequalities within the health care team by centralising decision-making power in the hands of medical profession at every level of care provision [6]. The powerlessness of nursing and midwifery leaders in health systems policy making, due to the doctor-centric nature of health policy making and regulation, has been a persistent challenge [8, 20].

The nursing councils do not play a role in regulation of practice, which is mainly managed by the state and central government bodies. Increasing the SNC’s role in practice regulation, with a new branch or a separate establishment for midwifery, will ensure quality and evidence-based nursing and midwifery care provision, which could be supervised by the INC to ensure uniformity [1]. Regulation is divided between different bodies including INC, the SNCs, the directorates of medical education and universities. The lack of clarity about this segregated nature of regulation adds to the confusion and decreases rigour when education and regulation is managed by so many different bodies, mostly without the involvement of nursing and midwifery representatives. It results in duplication of functions such as inspection; while other functions including the regulation of practice are completely ignored.

Literature suggests poor working condition and low remuneration as key drivers for nurse migration from India, which could be addressed by better regulation of practice [2, 19, 34]. Nurse (midwife) migration is also an area that needs to be regulated, given India is a major supplier of nurses to the Middle-East and high-income countries, ranking second in nurse outmigration after Philippines [8, 12, 40, 41]. Information about overseas migration and practice on return is provided in other country Acts [34, 42]. An understanding of the magnitude and reasons for nurse-migration will help to improve the quality of working conditions in India and decrease the workforce shortages by retaining more nurse-midwives.

The international experts interviewed presented a global perception mentioning some key challenges that were otherwise missed. The lack of evidence-based education was mentioned by a participant who felt the INC had a role to lead by showing best examples and guiding evidence-based education and practice in the country. They also highlighted the issue of underfunding for nursing and midwifery, from education to practice, and the development of professions and professionals.

**Fig 2:**
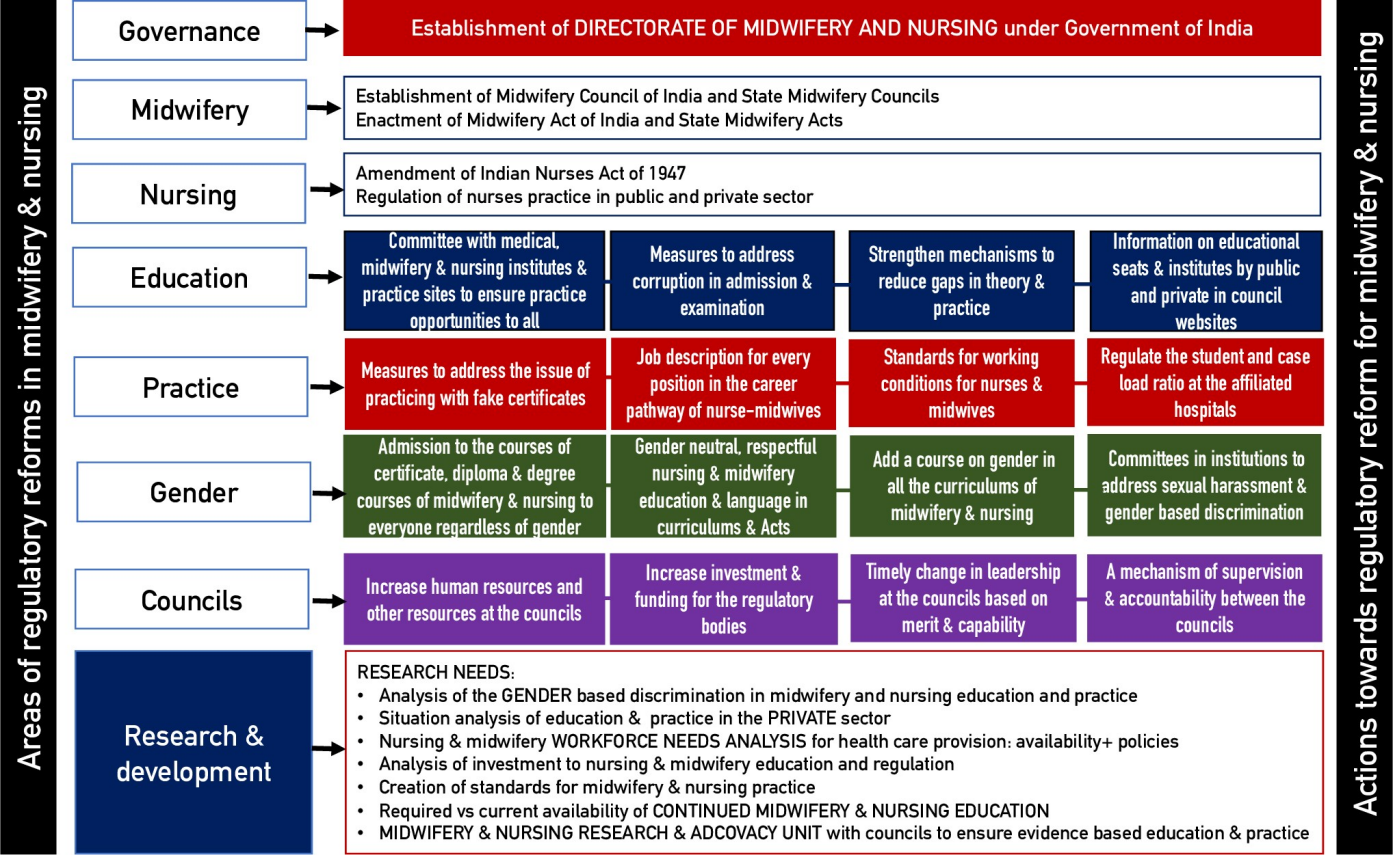
Needed areas for regulatory reforms in midwifery & nursing education & practice in India *(author’s original)*.

Similar challenges as shown in this study have been reported in relation to regulation of medical education, professionals and practice in India [43]. Although, the new National Medical Council (NMC) presents some scope for improvement, such as in the regulation of fees for medical education in private institutions [43], which is an area of reform for nursing and midwifery education as well along with regulation of salaries in the private sector. The regulations of fees is mentioned in the draft NNMC Bill, but the regulation of salaries in private sector is not [32]. The regulation of midwifery, nursing and medical education and practice are crucial for Universal Health Coverage (UHC). Fig 2 summarises areas and actions to reform midwifery and nursing, education and practice regulation in India along with the need for research in the future. It presents overarching areas of reform such as governance, where the establishment of midwifery and nursing directorate will aid many of the reforms suggested in the figure. Four key reform measures are suggested for each of the areas. There are some aspects which need more research and understanding in India, which also have been shown in Fig 2, such as, gender-based challenges in nursing, midwifery and health policy making; challenges in private sector education, regulation and practice; scope for independent midwifery practice; and implementation of innovative strategies in regulation to ensure good quality and respectful care provision.

## Conclusion

Nurses-midwives are the primary health care providers in India. Regulatory failures lead to nurses and midwives graduating without sufficient knowledge and skills, thereby putting lives and health at risk. This is a serious issue because practice is unregulated, care providers are unsupervised and not updated in a timely way while standards of care gradually deteriorate. The health regulatory structures of the country, including the regulatory bodies of all health care related professions, have a major role to play in maintaining standards of education and practice to ensure good quality of health care to its people. This requires a team approach similar to how a team of care providers with different expertise come together to provide quality health services. The INC, the SNCs, the Indian Medical Association (IMA), the directorates of medical education, public and private universities, nursing and midwifery associations and development organizations have a stake in health care regulation. These entities need to come together to understand the issues and work to address those challenges by creating a strong evidence based regulatory structure guided by midwifery and nursing leadership.

## Abbreviations

ANM: Auxiliary Nurse Midwife
ANMTC: Auxiliary Nurse Midwifery Training Centre
GNM: General Nursing and Midwifery
GNMTC: General Nursing and Midwifery Training Centre
GOI: Government of India
HRH: Human Resources for Health
ICM: International Confederation of Midwives
ICN: International Council of Nurses
INC: Indian Nursing Council
NHM: National Health Mission
NNMC Bill: National Nursing and Midwifery Commission Bill
NPM: Nurse Practitioner in Midwifery
NRTS: National Registration Tracking System
PSE: Pre Service Education
SNC: State Nursing Council
WHO: World Health Organization
